# Personalised relaxation practice to improve sleep and functioning in patients with chronic fatigue syndrome and depression: study protocol for a randomised controlled trial

**DOI:** 10.1186/s13063-018-2763-8

**Published:** 2018-07-11

**Authors:** Claire L. Macnamara, Erin Cvejic, Gordon B. Parker, Andrew R. Lloyd, Gina Lee, Jessica E. Beilharz, Ute Vollmer-Conna

**Affiliations:** 1 Department of Human Behaviour (Psychiatry), UNSW, Level 1, 30 Botany Street, Sydney, NSW 2052 Australia; 2 School of Public Health, University of Sydney, Sydney, Australia; 3 Black Dog Institute, Prince of Wales Hospital, Randwick, Sydney, Australia; 4 Viral Immunology Systems Program, The Kirby Institute, University of New South Wales, Sydney, Australia

**Keywords:** Chronic fatigue, Depression, Heart rate variability, Personalised intervention, Sleep

## Abstract

**Background:**

Chronic fatigue syndrome (CFS) and major depressive disorder (MDD) are both debilitating but heterogeneous conditions sharing core features of fatigue, unrefreshing sleep, and impaired functioning. The aetiology of these conditions is not fully understood, and ‘best-practice’ treatments are only moderately effective in relieving symptoms. Unrecognised individual differences in the response to such treatments are likely to underlie poor treatment outcomes.

**Methods/design:**

We are undertaking a two-group, parallel, randomised controlled trial (RCT) comparing the effects of a personalised relaxation intervention on sleep quality, daytime symptoms, and functioning in patients with CFS (*n* = 64) and MDD (*n* = 64). Following identification of the method that best enhances autonomic responding (such as heart rate variability), participants randomised to the active intervention will practise their recommended method nightly for 4 weeks. All participants will keep a sleep diary and monitor symptoms during the trial period, and they will complete two face-to-face assessments, one at baseline and one at 4 weeks, and a further online assessment to evaluate lasting effects of the intervention at 2 months. Assessments include self-report measures of sleep, wellbeing, and function and monitoring of autonomic responses at rest, in response to the relaxation method and during nocturnal sleep. Treatment outcomes will be analysed using linear mixed modelling.

**Discussion:**

This is the first RCT examining the effects of a personalised relaxation intervention, pre-tested to maximise the autonomic relaxation response, in patients with unrefreshing sleep and fatigue attributed to CFS or MDD. Detailed monitoring of sleep quality and symptoms will enable sensitive detection of improvements in the core symptoms of these debilitating conditions. In addition, repeated monitoring of autonomic functioning can elucidate mechanisms underlying potential benefits. The findings have translational potential, informing novel, personalised symptom management techniques for these conditions, with the potential for better clinical outcomes.

**Trial registration:**

Australian and New Zealand Clinical Trials Registry (ANZCTR), ACTRN12616001671459. Registered on 5 December 2016.

**Electronic supplementary material:**

The online version of this article (10.1186/s13063-018-2763-8) contains supplementary material, which is available to authorized users.

## Background

Chronic fatigue syndrome (CFS) is a debilitating disorder characterised by prolonged medically unexplained, disabling fatigue, with concomitant unrefreshing sleep, as well as other constitutional and neurocognitive symptoms [1]. Some of these core symptoms are shared by patients with major depressive disorder (MDD), notably fatigue, unrefreshing sleep, and neurocognitive difficulties [2, 3]. Both CFS and MDD are prevalent conditions [4, 5], yet despite international research efforts, their pathophysiological basis remains obscure, in part reflecting heterogeneity within the syndromal diagnoses [6, 7]. The disease burden and costs associated with these disorders are substantial both for the individual and society [8, 9], yet available management strategies for both conditions have only partial efficacy [10–12].

Autonomic nervous system (ANS) dysfunction is a common feature of a wide range of medical and neuropsychiatric conditions including CFS and MDD [13–15]. The ANS comprises sympathetic and parasympathetic divisions. Broadly, the sympathetic division is concerned with energy-demanding processes during times of activity and stress (e.g. the ‘fight-or-flight response’), whereas the parasympathetic division (notably via action of the vagus nerve) is associated with vegetative and restorative functions. These two divisions operate together to maintain homeostasis. The heart is dually innervated by both the excitatory sympathetic and inhibitory parasympathetic (vagal) divisions, resulting in constant flux of the interval between heartbeats. This variation in inter-beat interval is referred to as heart rate variability (HRV) and is widely regarded as a sensitive and reliable non-invasive method of assessing autonomic function [16–18]. Optimal autonomic function is characterised by flexible adaptation to environmental challenges and a marked increase in vagal activity during rest (reflected by high HRV), whereas low HRV and a shift towards sympathetic dominance reflect a vigilant, defensive physiological state lacking dynamic flexibility, suggestive of a system under stress [19].

There is a growing body of literature linking constitutionally reduced HRV with sleep difficulties in neuropsychiatric conditions including CFS and MDD [13, 20, 21]. Specifically, low HRV (which persists even during sleep) correlates strongly with the core symptoms of unrefreshing sleep, fatigue, and cognitive impairment in CFS [14, 15]. Similarly, somatic symptoms of depression such as fatigue and sleeping difficulties are more closely associated with lowered HRV than other symptoms of depression [22, 23]. In light of the evidence supporting a role for reduced HRV in the manifestation of debilitating symptoms, therapeutic interventions targeting autonomic imbalance may be beneficial to patients affected by these and potentially other fatigue-related conditions.

In the absence of curative treatments for CFS and MDD, interventions are aimed at symptom management and functional improvements [24–28]. These interventions include variants of physical activity programs such as graded exercise therapy (GET) and cognitive-behavioural therapy (CBT) [10, 29–31]. In addition to these therapies, behavioural interventions including relaxation and mindfulness-based approaches [32–34] are increasingly utilised, and more recently HRV biofeedback [35, 36] has been explored in CFS and MDD. The beneficial effects of such techniques, including improvements in autonomic functioning, are thought to be mediated via their impact on neural circuits involved in self-regulation and adaptation [19, 37, 38].

Despite their widespread use, these psycho-behavioural interventions typically show only small to moderate effect sizes, with average response rates rarely exceeding 30% [10, 12, 25, 31, 33, 39]. Drill-down analyses have revealed subgroups of patients who do not respond or who respond unfavourably to such interventions [40, 41]. Although it is recognised that individuals can differ considerably in their response to both pharmaceutical and psycho-behavioural therapies [11, 40, 42], individual variation in response to common therapies in fatiguing illnesses has not been examined in any depth.

We recently reported the first detailed study of individual differences in ANS responses to a variety of relaxation methods in healthy individuals [43]. We found that engagement with four different methods (soothing white noise, classical music, paced breathing, and guided relaxation) elicited significant differences in vagal activity (HRV, continuously monitored). Close inspection of the results revealed diversity in individual response patterns across the four relaxation methods. Indeed, the method that elicited the greatest HRV response overall varied significantly between participants. Moreover, there was no close association between participants’ subjective evaluation of the relaxation methods and the method that elicited their optimal HRV response. Thus, to maximise health outcomes, it could be beneficial to pre-test participants in order to determine their optimal method, rather than relying solely on subjective responses or, as is common practice, employing a ‘one-size-fits-all’ approach.

### Study objectives

Extending our novel findings, we are now conducting a randomised controlled trial (RCT) comparing the effects of a personalised intervention (determined from each participant’s best autonomic response to three different relaxation methods: guided relaxation, gentle noise, and instrumental music) to a sleep- and symptom-monitoring control condition in patients experiencing chronic fatigue due to CFS or MDD. This study aims to facilitate new approaches in clinical practice and more meaningful health outcomes for patients suffering from chronic fatigue states. The inclusion of two different patient groups with debilitating fatigue will provide additional insights into the generalisability of benefits derived from such an intervention to other groups with fatiguing illnesses. The specific aims of this clinical trial are to:

1. Determine the efficacy and specific benefits of a 4-week personalised relaxation intervention in terms of (a) subjective health outcomes and (b) restoration of autonomic balance in patients with CFS, and with MDD, compared to a sleep- and symptom-monitoring control condition
2. Perform a follow-up assessment 4 weeks after the completion of the intervention to document enduring health-related benefits that flow from participation in a personalised relaxation intervention (active group only).

It is hypothesised that the personalised relaxation intervention, pre-tested to maximise the participant’s autonomic relaxation response, will be substantively more effective in improving sleep quality as well as daytime fatigue, symptoms, and functioning than sleep and symptom monitoring only (no relaxation intervention). Additionally, it is expected that nightly practice of the intervention will improve autonomic functioning in patients suffering from both depression and fatigue. The findings from this research will facilitate a better understanding of the pathophysiological mechanisms operating in chronic fatigue states.

## Methods/design

### Study design

**Fig. 1:**
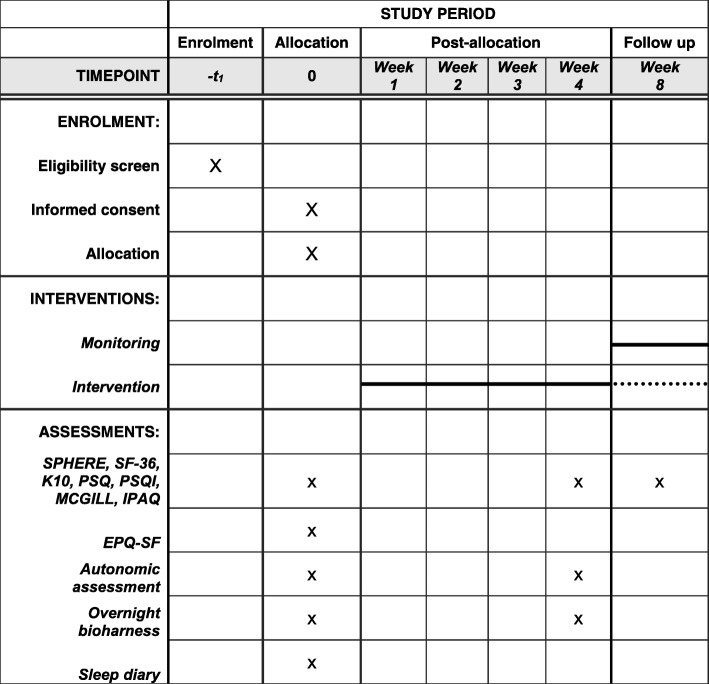
Schedule of enrolment, interventions, and assessments (Standard Protocol Items: Recommendations for Interventional Trials (SPIRIT) diagram)

This study is a two-parallel group RCT comparing a personalised relaxation intervention to a sleep- and symptom-monitoring control condition. All assessments are conducted at the University of New South Wales Sydney (UNSW), Australia. The trial has been registered on the Australian and New Zealand Clinical Trials Registry (ANZCTR, ACTRN12616001671459). All dimensions of the study protocol have been described adhering to the Standard Protocol Items: Recommendations for Interventional Trials (SPIRIT) checklist (Additional file 1; Fig. 1).

### Sampling and recruitment

Potential participants are screened by email or telephone to ensure they meet eligibility criteria before proceeding to enrolment. Individuals are eligible to participate if they are aged between 18 and 65 years at the time of assessment; have been diagnosed by a physician or psychiatrist to meet international diagnostic criteria for CFS [1] or MDD with fatigue as a significant feature as defined by Diagnostic and Statistical Manual of Mental Disorders, 5^th^ edition (DSM-V) diagnostic criteria [44]; have self-reported normal hearing; sufficient English language proficiency to complete questionnaires and understand instructions; and a willingness to participate and comply with the longitudinal nature of the study. Individuals who are concurrently engaged in relaxation or mindfulness-based therapies; are pregnant; have other significant illnesses or major diagnoses such as primary sleep disorder, heart conditions, uncontrolled diabetes, chronic infections, or psychotic disorders; or who take regular medications that affect autonomic activity, including beta blockers and anti-hypertensives, are excluded from the study. Use of anti-depressant medication or hormonal contraception is recorded, but is not exclusionary.

Patients with CFS are recruited from an academic tertiary referral clinic which specialises in the management of chronic fatigue states (UNSW Fatigue Clinic in Sydney, Australia) via advertisements placed in clinic waiting rooms and targeted email advertisement distribution to the clinic’s opt-in volunteer research register. Participants with MDD are recruited from the Black Dog Institute (BDI), a mental health research centre and clinic in Sydney, via online advertisements and email distribution to the BDI volunteer research register.

### Randomisation, allocation, and blinding

A permuted block randomisation sequence (block sizes of four), stratified by clinical condition, was generated using Stata 13 by the study biostatistician (EC) prior to trial commencement. The randomisation sequence is stored in a password-protected file not accessible to the researcher responsible for enrolling and assessing participants. Treatment allocation remains concealed until immediately before a participant’s first assessment, at which time the assessing researcher is informed of the allocation by the study administrator. Due to the nature of the intervention, both the participant and assessing researcher are not blinded to group assignment. During primary data analysis, the biostatistician will be blinded to treatment group (i.e. by using unlabelled numerical coding for the treatment group variable within the dataset, recoverable by the study administrator).

### Sample size estimates

Statistical power and appropriate effect size estimates are based on published recommendations [45] and unpublished pilot data using this protocol collected by our group. A sample size of 128 participants (64 in each treatment arm) provides ≥ 80% power (with a Bonferroni-adjusted significance level of 0.013 allowing for multiple comparisons) to detect improvements of approximately 1 standard deviation in sleep quality (2-point reduction), fatigue (3-point reduction), and functioning (8-point improvement) measures in the intervention arm [46]. This conservative estimate will allow for an attrition rate of up to 10%.

### Data collection

**Fig. 2:**
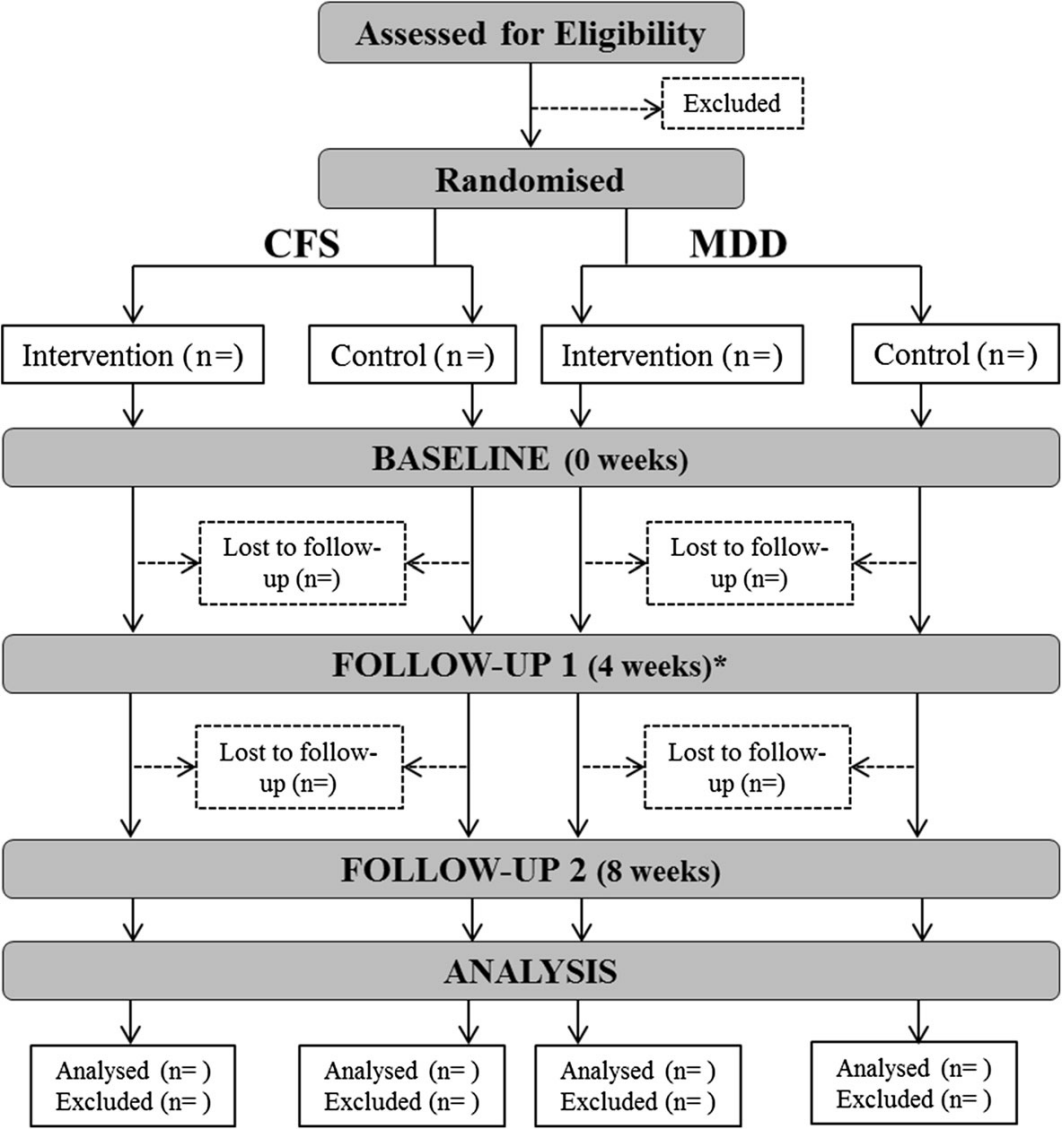
Flow diagram of participant enrolment, assessment, and analysis. *Control participants were offered their personalised relaxation intervention

All consenting participants complete two face-to-face assessments, 4 weeks apart, and a further set of questionnaires completed online 8 weeks after the initial assessment (Fig 2). At the first assessment (lasting approximately 90 min), participants’ demographic, health, and symptom information are documented. Autonomic activity at rest and in response to each of the relaxation methods (see below) is recorded. Results from this initial testing determine which method participants randomised to the intervention group will practise daily during the 4-week intervention (i.e. the method which induced the greatest increase in HRV for that participant). Before leaving, all participants are fitted with a non-invasive ambulatory bioharness (Equivital EQ-02, Hidalgo Ltd., Cambridgeshire, UK) for 24-h monitoring and issued a sleep/activity/symptom log to complete over the next 4 weeks (see subsequent paragraphs). The second assessment lasts approximately 45 min and consists of a briefer version of the initial assessments (i.e. assessment of health, symptoms, and autonomic activity). Those in the control arm will be offered the personalised intervention at this assessment. The third assessment consists of online self-report measures, including questionnaires about the participant’s sleep, symptoms, and current frequency of relaxation practice. This will allow us to assess possible enduring benefits of the intervention in the active group, and to determine whether subjective improvements are evident in the monitoring group who will have practised their assigned intervention for 4 weeks. Participants will be emailed a personalised link to the online questionnaire. An automated reminder email will be sent after 1 week and again after 2 weeks, if the participant has not completed the questionnaire. The online nature of this assessment will reduce inconvenience for the participants and hence minimise attrition.

### Assessment measures

#### Self-report measures

Commonly used, standardised and validated questionnaires were selected to evaluate symptom domains relevant to sleep, as well as fatigue, other symptoms, and functioning relevant to the two patient groups suffering from CFS and MDD. The Somatic and Psychological Health Report (SPHERE) [47] is used to assess current somatic and psychological symptoms. Items are rated on a scale of 0–2 (0 = never troubled, 2 = troubled most of the time); this instrument produces two subscales (SOMA and PSYCH), both of which have high internal consistency (PSYCH 0.90 [Cronbach’s alpha], SOMA 0.80) and test-retest reliability (PSYCH 0.81, SOMA 0.80). Psychological distress is assessed via the Kessler Psychological Distress Scale (K10; consisting of 10 items, each rated between 1 = none of the time and 5 = all of the time); reported reliability for the K10 scale is high alpha = 0.93 [48]. The Perceived Stress Questionnaire (PSQ) consists of 30 items, each rated between 1 = almost never and 4 = usually); reliability values reported for PSQ include 0.90 for internal consistency and 0.82 for test-retest reliability [49]. The 24-item Pittsburgh Sleep Quality Index (PSQI) [50] is used to assess overall sleep quality. The overall reliability coefficient for PSQI is 0.83. A global PSQI score > 5 yields a diagnostic sensitivity of 89.6% and a specificity of 86.5% (kappa = 0.75, *p* < 0.001) in distinguishing good and poor sleepers. Pain levels are measured using the short-form McGill Pain Questionnaire; the scale consists of 15 descriptors (11 sensory; 4 affective) which are rated on an intensity scale as 0 = none, 1 = mild, 2 = moderate, or 3 = severe. Three pain scores are derived from the sum of the intensity rank values of the words chosen for sensory, affective, and total descriptors. Cronbach’s alpha for a total scale reliability score is reported as 0.92 for older and 0.89 for younger participants [51].

The personality traits neuroticism and extroversion are quantified using the two subscales of the Eysenck Personality Questionnaire Short form (EPQ-SF) [52]. Each of these subscales consists of 12 questions that require a ’yes’ or ’no’ answer. The total score for each subscale is 12, with a higher score indicating higher trait attributes. Cronbach’s alpha values for males and females are 0.84 and 0.80 respectively for the neuroticism scale, and 0.88 and 0.84 respectively for the extroversion scale. Physical activity is documented using the International Physical Activity Questionnaire (IPAQ) Short Form [53]. The questionnaire consists of four questions on time spent in walking, vigorous and moderate-intensity activity, and sedentary activity, where vigorous activities include heavy lifting or fast bicycling, and examples of moderate activities are carrying light loads or bicycling at a regular pace. For each of these activity types the average time usually spent on each is recorded for ‘the last 7 days’, and total scores are calculated with consideration of the energy expenditure for each activity category. Spearman correlation coefficients, reported to indicate test-retest reliability, were at an acceptable level, with 75% of the correlation coefficients observed above 0.65 [53].

The Medical Outcomes Survey Short Form-36 (MOS SF-36) is a 36-item questionnaire used to measure aspects of health and functional disability across eight domains including physical functioning, role limitations due to physical health, role limitations due to emotional problems, energy/fatigue, emotional wellbeing, social functioning, pain, and general health. Eight scaled scores are derived, which are the weighted sums of the questions in their domain. Each score is directly transformed into a 0–100 scale on the assumption that each question carries equal weight. A lower score indicates more disability/less health. Internal consistency reliability estimates of the eight scales are reported to range between 0.73 to 0.96, with a median of 0.95; median estimates for test-retest reliability were 76 [54]. All participants are asked to complete these questionnaires at all assessments, including baseline and follow-up at 4 weeks and for the online assessment. The EPQ-SF is only administered at the baseline assessment.

After the presentation of each relaxation method, participants are asked how relaxing they found the method (on a scale ranging from ‘not at all’ to ‘very’ relaxing) and whether they would expect the intervention to be helpful in reducing their symptoms (‘no’, ‘maybe’, ‘yes’). At the second assessment, participants in the intervention group are asked if they were satisfied with the intervention in improving their symptoms (on a scale ranging from ‘not at all’ to ‘completely’). At the 8-week online assessment, participants are asked if they have continued to practise their assigned relaxation method, and if so, how frequently.

#### Autonomic assessment

Laboratory-based autonomic measures include three-lead electrocardiogram (ECG) and respiration (via a strain gauge transducer) recorded at 1 kHz using PowerLab and LabChart Pro 8 (ADInstruments, Bella Vista, Australia). To obtain a baseline assessment of cardiac autonomic functioning, participants are asked to relax in a semi-reclined position for 10 min, in silence. Autonomic responses are also measured during the presentation of each of the three relaxation methods (each ~ 10-min duration). Immediately after the assessment, data are processed and analysed using the LabChart HRV 2.0 module to determine the participant’s baseline HRV and his/her response during each of the relaxation methods to allow selection of the method that induced the largest increase in HRV response for that participant.

Continuous 24-h monitoring of autonomic activity (including during nocturnal sleep) is achieved via a lightweight bioharness system (Equivital, Hidalgo) at baseline and at 4 weeks. Participants are fitted with a non-invasive chest harness consisting of a two-channel ECG (sampling rate 256 Hz), respiratory belt (25.6 Hz), skin temperature sensor (25.6 Hz), and tri-axial accelerometer (25.6 Hz, enabling detection of body orientation and movement). The overnight data are extracted and processed using LabChart Pro.

#### Relaxation methods

Immediately following the resting baseline assessment, participants are presented with an example of three relaxation methods: Guided Relaxation, Gentle Noise, and Instrumental Music, with the order counter-balanced across participants. The Guided Relaxation method consists of a calming voice talking through body awareness and progressive relaxation. The Gentle Noise method uses a soft, undulating white noise which sounds similar to the ocean or rain. The Instrumental Music method uses a calming musical piece (Mozart’s Flute and Harp Concerto in C, K.299 2nd Movement). Participants listen to each of these methods through headphones, at a comfortable listening volume, whilst in a semi-reclined position. For each method, participants are instructed to clear their thoughts, relax, breathe normally, and, for the guided piece, to follow the verbal directions.

#### Sleep, activity, and symptom log

Throughout the 4-week trial period, all participants keep a brief log of their sleep, activity (every day), symptoms (once per week, SOMA subscale [47] of the SPHERE questionnaire), and psychological distress (once per week, K10 [48]). Those in the intervention arm also record the amount of time spent practising their assigned method every day. Participants are contacted weekly via their preferred method, either email or telephone, to maximise engagement and compliance.

### Intervention

#### Personalised relaxation intervention

Participants allocated to the intervention are assigned to practise the method which generated the greatest relaxation response (as quantified by an increase in HRV) relative to their resting baseline, thus optimising de-arousal.

Participants have free online access to their assigned method and are able to download the recordings (10-min segments) to their smartphone or media device, or if preferred a portable media player is provided. A number of similar variants of their assigned method are offered to maximise enjoyment and prevent negative effects of frequent repetition. Participants are instructed to practise their assigned method at a minimum before bedtime every evening for 4 weeks, but may use the method at other times if desired.

#### Sleep and symptom monitoring

Participants in the control condition complete the same assessments as the intervention group at baseline and at the 4 week follow-up. Control participants are informed that 4 weeks of symptom monitoring will precede allocation of a personalised relaxation method. During this period they are asked not to practise mindfulness or meditation activities. At the completion of the second assessment, participants in the control group will be offered their personalised intervention.

### Outcomes

The primary outcomes for this study are changes in self-reported sleep quality, symptoms, and functioning. These will be assessed by self-report measures at the 4-week assessment. Secondary outcomes are changes in HRV and interventional acceptability.

### Data analysis plan

The outcome variable (change scores) will be analysed using linear mixed modelling. Treatment arm (intervention/control) and clinical condition (MDD/CFS) will be included in models as a dichotomous between-subjects variable. Baseline values of the outcome variable will also be included as a covariate (to control for chance differences between treatment arms at baseline). Bivariate associations between demographic measures and outcome variables will be explored using Pearson pairwise correlations. The outcome of these analyses will inform about additional variables to be included in multivariate models as covariates.

## Ethics and dissemination

Potential participants are informed that their decision of whether or not to participate will not affect their relationship with the UNSW Fatigue Clinic or the BDI. All participants provide written informed consent before commencing any assessments and are free to withdraw at any time without consequence (see Additional file 2). Participants are given a signed consent form to take home which also includes information about the study and the contact details of a trained clinician, and a telephone helpline *beyondblue*. Participants can call these numbers in the unlikely event that they become distressed from the study and require support from someone not involved in any direct assessments. Participants are encouraged to respond in an open and honest manner to all questionnaires and are reassured that their participation and the responses they provide will remain confidential and have no bearing on their involvement in the study or on their current or future treatment.

All response data are entered into a secured computer database. Only research staff directly involved with the project has access to the physical and electronic data. Upon enrolment, participants are assigned a unique study identification number which is used across all study materials (questionnaires and computer files), allowing data to be linked during analysis without revealing the identity of the individual participants. To maintain anonymity of participants, only de-identified data, presented as group means and differences, are used in all forms of dissemination. In line with National Health and Medical Research Council (NHMRC) recommendations, all data will be kept for a minimum of 15 years. At time of disposal, hard-copy data will be shredded, and electronic media will be erased and re-formatted.

The findings from this study will be disseminated through international peer-reviewed journals and presentations at relevant national and international conferences. Participants who indicated on their consent form an interest in the overall results of the study will be sent (via email or post) a lay summary of the findings. If the results of the trial point towards beneficial effects of the intervention, the online links to the relaxation methods will be distributed to relevant stakeholders such as the UNSW Fatigue Clinic and the BDI.

## Discussion

Patients with CFS or MDD frequently experience unrefreshing sleep and debilitating fatigue, which have negative consequences for daytime functioning [1, 3]. Although the pathophysiology of these conditions is not well understood, ANS dysfunction is evident in both patient groups and may influence symptoms [13, 55]. Current management strategies, including CBT, exercise programs, and relaxation methods, are only moderately successful and do not always deliver beneficial outcomes [10, 12, 25, 31, 33, 39]. Abandoning a ‘one-size-fits-all’ approach and moving towards personalised treatment options may offer another way forward.

To the best of our knowledge, this is the first RCT to prescribe a personalised intervention based on the participant’s maximal autonomic relaxation response. We propose that using such an approach will lead to more substantial improvements in sleep quality, reduced daytime fatigue and other symptoms, and enhanced functional status. The inclusion of two patient groups with fatiguing illness due to CFS or MDD will provide insights into generalisability of the potential health benefits resulting from personalised relaxation intervention to other relevant patient populations, thus maximising translational utility.

## Trial status

The trial was registered on 5 December 2016 (ACTRN12616001671459). Study recruitment commenced in April 2017 and will continue for 2 years or until the required sample size is reached.

## Additional files

Additional file 1: Standard Protocol Items: Recommendations for Interventional Trials (SPIRIT) 2013 Checklist: recommended items to address in a clinical trial protocol. (DOC 122 kb)

Additional file 2: Participant informed consent form. (DOCX 1310 kb)

## Abbreviations

ANS: Autonomic nervous system
ANZCTR: Australian and New Zealand Clinical Trials Registry
BDI: Black Dog Institute
CBT: Cognitive-behavioural therapy
CFS: Chronic fatigue syndrome
ECG: Electrocardiogram
EPQ-SF: Eysenck Personality Questionnaire Short Form
GET: Graded exercise therapy
HRV: Heart rate variability
IPAQ: International Physical Activity Questionnaire
K10: Kessler Psychological Distress Scale
MDD: Major depressive disorder
MOS SF-36: Medical Outcomes Survey Short Form-36
PSQ: Perceived Stress Questionnaire
PSQI: Pittsburgh Sleep Quality Index
RCT: Randomised controlled trial
SPHERE: Somatic and Psychological Health Report
UNSW: University of New South Wales Sydney
