# Supplementary material for: Personalised relaxation practice to improve sleep and functioning in patients with chronic fatigue syndrome and depression: study protocol for a randomised controlled trial
Source: Trials. 2018 Jul 11;19:371. doi: 10.1186/s13063-018-2763-8 (PMC6042263; doi:10.1186/s13063-018-2763-8)
Supplement: Supplementary file 2 — Participant informed consent form. (DOCX 1310 kb) [file 13063_2018_2763_MOESM2_ESM.docx]

| This research is being carried out by the following researchers: | | |
| --- | --- | --- |
| ***Role*** | ***Name and Contact Number*** | ***Organisation*** |
| **Chief Investigator** | A/Prof. Ute Vollmer-Conna (Ph: 02 9385 2945) | School of Psychiatry, UNSW &  The Black Dog Institute |
| **Co-Investigators** | Dr. Erin Cvejic  Scientia Prof.Gordon Parker  Prof. Andrew Lloyd  Ms. Claire Macnamara  Ms. Jessica Beilharz (Ph: 02 9385 2942) | School of Psychiatry, UNSW  School of Psychiatry, UNSW  The Kirby Institute, UNSW  School of Psychiatry, UNSW |
| **Research Funder** | This research is being funded by The Mason Foundation (RG152203) | |

**What is the research study about?**

This study aims to examine the effect of a 4 week personalised intervention (individually selected for each participant to generate the best relaxation response) on improving sleep quality and daytime functioning in patients with chronic fatigue syndrome (CFS) and depression. You have been invited to participate because you are a patient with CFS or depression, who has contacted us and have expressed interest in participating.

**Inclusion/Exclusion Criteria**

To participate in this project you need to meet the following criteria:

- Aged between 18 and 65 years;
- No significant illness (other than chronic fatigue syndrome or depression), such as heart conditions, uncontrolled diabetes, chronic infections, psychotic disorders, or primary sleep disorders;
- No significant hearing impairment;
- Not pregnant
- Not taking any regular medications that affect autonomic activity such as beta-blockers/anti-hypertensive. Use of anti-depressants or the oral contraceptive pill is not exclusionary.

**Do I have to take part in this research study?**

This Participant Information Statement and Consent Form tells you about the study. It explains the research tasks involved. Knowing what is involved will help you decide if you want to take part in the research. Please read this information carefully. Ask questions about anything that you don’t understand or want to know more about. Before deciding whether or not to take part, you might want to talk about it with a relative or friend.

If you decide you want to take part in the study, you will be asked to:

- Sign the consent form;
- Keep a copy of this Participant Information Statement;

Participation in this study is voluntary. If you don’t wish to take part, you don’t have to. Your decision will not affect your relationship with The University of New South Wales or The Black Dog Institute.

**What does participation in this research require, and are there any risks involved?**

If you decide to take part in the research study, we will ask you to complete the following tasks across two assessment visits and an online questionnaire. The order of these activities and their associated risks are detailed in the study visit table on page 3.

- **Self-report questionnaires**

You will be asked to complete a number of paper-based questionnaires regarding your demographic, physical and mental health, and lifestyle information, and current symptoms that you may be experiencing.

- **Autonomic assessment during relaxation methods**

Your heart rate, breathing rate, and skin sweat response will be recorded using non-invasive equipment. We will first ask you to rest for 10 minutes while we record a baseline measurement. You will then be presented with three relaxation methods approximately 10 minutes each (i.e. gentle noise, instrumental music and guided relaxation) in random order, during which time your autonomic response will be recorded.


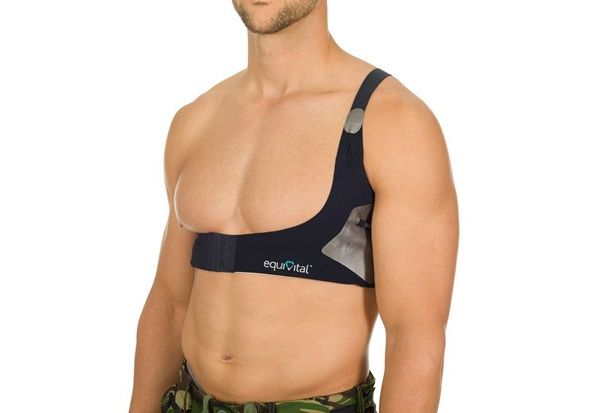


- **Overnight autonomic recording**

Before leaving the laboratory, you will be fitted with a lightweight, portable heart rate monitor to wear overnight until the following morning, including during sleep. This device will continuously record your heart rate, respiration and sleep-wake behaviour (time spent moving and orientation). The device is designed to maximise comfort and looks similar to a sports bra (as shown in the picture to the right), and is worn under your clothing. We will organise a courier to come and collect the device from a convenient location and at no cost to you on the following day.

- **Personalised relaxation practice**

You will be provided with access to pre-recorded 10-minute segments of a relaxation method for download onto your phone or portable media device. You will be asked to practice this method in your own home daily for 4 weeks, for a minimum of 10 minutes before bedtime and may use at other times if you feel comfortable. You will also be asked to keep a brief log of your sleep, activity and symptoms during this 4-week period. Participants will undertake relaxation practice immediately, or after a four week monitoring period.

- **Repeated assessments**

You will be asked to return to the lab after the 4-week practice and monitoring period for reassessment, which will involve completing a briefer set of self-report questionnaires and autonomic monitoring (at rest, during presentation of your optimal relaxation method, and overnight in your own home). We expect this second visit to take approximately 45 minutes. One month after the second visit a brief online questionnaire will be emailed to you for completion.

Aside from giving up your time, we do not expect that there will be any additional risks or costs associated with taking part in this study.

**What are the possible benefits to participation?**

Although we cannot guarantee any personal benefits from taking part in this research project, the results could lead to a better understanding of the underlying causes of unrefreshing sleep and impaired daytime functioning in patients with chronic fatigue syndrome and depression, and contribute to the development of non-pharmacological treatments for patients experiencing these conditions.

**What will happen to information about me?**

By signing the consent form you consent to the research team collecting and using information about you for the research study. We will keep your data for a period of 7 years. We will store information about you in a non-identifiable format at 30 Botany Street, UNSW. Your information will only be used for the purpose of this research study and it will only be disclosed with your permission.

| **Study visit table** | | | |
| --- | --- | --- | --- |
| ***Day / Location*** | ***Activity*** | ***Risk (if applicable)*** | ***Time Commitment*** |
| **Day 1**  **(Visit 1)**  Autonomic Assessment Lab, UNSW  *~90 mins* | Self-report questionnaires | Although highly unlikely, you may feel that some of the questions asked are stressful or upsetting. If you do not wish to answer a question in the questionnaires, you may skip it and go to the next question, or you may stop immediately. If you become upset or distressed as a result of participation in the research project, the research team will be able to arrange for counselling or other appropriate support for you free of charge. | 30 minutes |
|  | Heart rate and breathing monitored non-invasively during relaxation methods | All monitoring equipment is non-invasive, and involves no risk. Some participants may be anxious about the completion of the relaxation methods, although this is highly unlikely. If you find any of the tasks too demanding or worrying, you are free to withdraw at any time without consequence. | 40 minutes |
|  | Fitted with portable heart rate monitor / issued with sleep log | The monitoring equipment is non-invasive, and involves no risks. There is a small possibility of developing a rash from where the monitoring belt sits on the body, but this is highly unlikely. | 10 minutes |
| **Day 2** | *Ambulatory monitor collected by courier from convenient location* | | |
| **Day 2- Day 28**  Own home | Practice assigned mindfulness-based activity / complete sleep, activity, and symptom log | Participants may develop anxieties about the completing the required amount of relaxation practice, although this is highly unlikely. If you find that performing the practice is too difficult, you are free to withdraw at any time without consequence. | 11 minutes |
| **Day 29**  **(Visit 2)**  Autonomic Assessment Lab, UNSW | As per Visit 1 | As above | 45 minutes |
| **Day 30** | *Ambulatory monitor collected by courier from convenient location* | | |
| **Day 60**  Own home | Self-report questionnaires (online) | As above | 20 minutes |

**How and when will I find out what the results of the research study are?**

The research team intend to publish and/ report the results of the research study in a variety of ways. All information published will be done in a way that will not identify you. If you would like to receive a copy of the results you can let the research team know by adding your email or postal address within the consent form. We will only use these details to send you the results of the research at the conclusion of the study. This will be in the form of one page summary of the research findings. Personalised feedback will not be provided.

**What if I want to withdraw from the research study?**

If you do consent to participate, you may withdraw at any time. You can do so by completing the ‘Withdrawal of Consent Form’ which is provided at the end of this document. Alternatively you can ring the research team and tell them you no longer want to participate. If you decide to leave the research study, the researchers will not collect additional information from you. Please let us know at the time when you withdraw what you would like us to do with the information we have collected about you up to that point. If you wish your information will be removed from our study records and will not be included in the study results, up to the point that we have analysed and published the results. Your decision to withdraw will not affect your relationship with UNSW Australia or the Black Dog Institute.

**What should I do if I have further questions about my involvement in the research study?**

The person you may need to contact will depend on the nature of your query. If you want any further information concerning this project or if you have any problems which may be related to your involvement in the project, you can contact the following member/s of the research team:

**Research Team Contact Details**

| **Name** | Ms Jessica Beilharz |
| --- | --- |
| **Position** | Research Assistant, School of Psychiatry, UNSW |
| **Telephone** | 02 9385 2942 |
| **Email** | [human.behav@unsw.edu.au](mailto:human.behav@unsw.edu.au) and include “Relaxation Study” in the email title |

**Support Services Contact Details**

If at any stage during the project you become distressed or require additional support from someone not involved in the research please call:

| **Name/Organisation** | Uté Vollmer-Conna |
| --- | --- |
| **Position** | Associate Professor in Psychiatry, UNSW / Registered Psychologist |
| **Telephone** | 02 9385 2945 |
| **Email** | [ute@unsw.edu.au](mailto:ute@unsw.edu.au) |

If you would prefer to contact a trained mental health professional external to the research project about general feelings of distress, *beyondblue* offers a confidential telephone support service available 24 hours a day, 7 days a week, to listen, provide information and advice, and point you in the right direction so that you can seek further support if necessary.

| **Name/Organisation** | beyondblue Support Service |
| --- | --- |
| **Telephone** | 1300 22 46 36 |

**What if I have a complaint or any concerns about the research study?**

If you have any complaints about any aspect of the project, the way it is being conducted, then you may contact:

**Complaints Contact**

| **Position** | Human Research Ethics Coordinator |
| --- | --- |
| **Telephone** | + 61 2 9385 6222 |
| **Email** | [humanethics@unsw.edu.au](mailto:humanethics@unsw.edu.au) |
| **HC Reference Number** | HC16953 |

**Consent Form – Participant providing own consent**

**Declaration by the participant**

- I understand I am being asked to provide consent to participate in this research project;
- I have read the Participant Information Sheet or someone has read it to me in a language that I understand;
- I understand the purposes, study tasks and risks of the research described in the project;
- I provide my consent for the information collected about me to be used for the purpose of this research study only.
- I have had an opportunity to ask questions and I am satisfied with the answers I have received;
- I freely agree to participate in this research study as described and understand that I am free to withdraw at any time during the project and withdrawal will not affect my relationship with any of the named organisations and/or research team members;
- I understand that I will be given a signed copy of this document to keep;
- I would like to be informed about the outcomes of this study, and be sent a summary of the findings to the following address (post or email):

_______________________________________________________________________

_______________________________________________________________________

**Participant Signature**

| Name of Participant (please print) |  |
| --- | --- |
| Signature of Research Participant |  |
| Date |  |

**Declaration by Researcher***

- I have given a verbal explanation of the research study, its study activities and risks and I believe that the participant has understood that explanation.

**Researcher Signature***

| Name of Researcher (please print) |  |
| --- | --- |
| Signature of Researcher |  |
| Date |  |

**^+^An appropriately qualified member of the research team must provide the explanation of, and information concerning the research study.**

**Note: All parties signing the consent section must date their own signature.**

**Form for Withdrawal of Participation**

I wish to **WITHDRAW** my consent to participate in the research proposal described above and understand that such withdrawal **WILL NOT** affect my relationship with The University of New South Wales or the Black Dog Institute.

**Participant Signature**

| Name of Participant  (please print) |  |
| --- | --- |
| Signature of Research Participant |  |
| Date |  |

**The section for Withdrawal of Participation should be forwarded to:**

| CI Name: | A/Prof. Ute Vollmer-Conna |
| --- | --- |
| Email: | [ute@unsw.edu.au](mailto:ute@unsw.edu.au) |
| Phone: | 02 9385 2945 |
| Postal Address: | Department of Human Behaviour (Psychiatry)  Level 1, 30 Botany Street  UNSW SYDNEY, NSW 2052 |
